# Bim, Puma and Noxa upregulation by Naftopidil sensitizes ovarian cancer to the BH3-mimetic ABT-737 and the MEK inhibitor Trametinib

**DOI:** 10.1038/s41419-020-2588-8

**Published:** 2020-05-18

**Authors:** Romane Florent, Louis-Bastien Weiswald, Bernard Lambert, Emilie Brotin, Edwige Abeilard, Marie-Hélène Louis, Guillaume Babin, Laurent Poulain, Monique N’Diaye

**Affiliations:** 1 Normandie Univ, UNICAEN, INSERM U1086 ANTICIPE (Interdisciplinary Research Unit for Cancers Prevention and Treatment), BioTICLA Axis (Biology and Innovative Therapeutics for Ovarian Cancers), Caen, France; 2 UNICANCER, Cancer Center François Baclesse, Caen, France; 3 CNRS—Regional Delegation of Normandy, Caen, France; 4 Biological Ressources Center «OvaRessources», Cancer Center François Baclesse, Caen, France

**Keywords:** Ovarian cancer, Apoptosis

## Abstract

Ovarian cancer represents the first cause of mortality from gynecologic malignancies due to frequent chemoresistance occurrence. Increasing the [BH3-only Bim, Puma, Noxa proapoptotic]/[Bcl-x_L_, Mcl-1 antiapoptotic] proteins ratio was proven to efficiently kill ovarian carcinoma cells and development of new molecules to imbalance Bcl-2 member equilibrium are strongly required. Drug repurposing constitutes an innovative approach to rapidly develop therapeutic strategies through exploitation of established drugs already approved for the treatment of noncancerous diseases. This strategy allowed a renewed interest for Naftopidil, an α_1_-adrenergic receptor antagonist commercialized in Japan for benign prostatic hyperplasia. Naftopidil was reported to decrease the incidence of prostate cancer and its derivative was described to increase BH3-only protein expression in some cancer models. Based on these arguments, we evaluated the effects of Naftopidil on ovarian carcinoma and showed that Naftopidil reduced cell growth and increased the expression of the BH3-only proteins Bim, Puma and Noxa. This effect was independent of α_1_-adrenergic receptors blocking and involved ATF4 or JNK pathway depending on cellular context. Finally, Naftopidil-induced BH3-only members sensitized our models to ABT-737 and Trametinib treatments, in vitro as well as ex vivo, in patient-derived organoid models.

## Introduction

Ovarian cancer represents the first cause of mortality from gynecologic malignancies in United States and Europe^1^ with a survival rate at 5 years not exceeding 30% for patients with late-stage disease^2^. The bad prognosis of this pathology is mainly due to a poor symptomatology, which delays its discovery and leads to diagnosis at advanced stages^3^. Although a good initial response to conventional chemotherapy, a majority of patients will experience relapses and development of treatment resistance leading to death^4^.

Expression of members of the Bcl-2 family is frequently disturbed during carcinogenesis^5^ and overexpression of antiapoptotic factors is often associated with chemoresistance^6^. Upon oncogenic stress, the synthesized BH3-only proteins are then trapped by the excess of antiapoptotic molecules impeding apoptosis induction^7^. In this context, we demonstrated that the concomitant inhibition of Bcl-x_L_ and Mcl-1 is sufficient to induce apoptosis in chemoresistant ovarian cell lines in vitro^8^ and increasing the [proapoptotic BH3-only Bim, Puma, Noxa]/[antiapoptotic Bcl-x_L_, Mcl-1] protein ratio by upregulating proapoptotic proteins or inhibiting antiapoptotic members constitutes a therapeutic opportunity to trigger apoptotic cell death.

Disrupting antiapoptotic proteins to their BH3-only partners could be achieved by using BH3-mimetic molecules that hinder the function of antiapoptotic proteins by binding their hydrophobic groove^9^. Among them, ABT-263 (Navitoclax), the orally administrable derivative of ABT-737, is currently undergoing phase II clinical trials in different locations including ovarian cancer (NCT02591095). However, ABT-737 does not target Mcl-1 and inhibiting Mcl-1 and/or inducing its BH3-only partners (Bim, Puma and Noxa) represent a rational strategy to sensitize ovarian carcinoma cells to ABT-737 ^10,11^. BH3-only proteins were described to be regulated through the activation of Akt/mTOR and MAPK/ERK survival pathways and inhibitors of these pathways have proven to efficiently sensitize to ABT-737 ^12,13^. As stress sensors, BH3-only are also known to be upregulated by many molecular pathways including endoplasmic reticulum (ER) stress^14–17^, c-Jun NH2-terminal kinase (JNK)^18–20^, nuclear factor-κB (NFκB)^17,21–23^ or reactive oxygen species (ROS) accumulation^24–26^. Targeting these transduction pathways could be considered as an indirect way of unleashing BH3-only apoptotic activity and overcoming antiapoptotic proteins buffering capacity.

An innovative approach to rapidly develop efficient therapeutic strategies is the exploitation of established drugs already approved for the treatment of noncancerous diseases. This strategy, also called drug repurposing or therapeutic switching, presents the main advantage to “recycle” molecules that pharmacokinetic, pharmacodynamic, and toxicity profiles are well known, promoting their rapid translation into clinical trial^27^. This strategy allowed a renewed interest for α_1_-adrenergic receptor (α_1_-AR) antagonists as anticancer agents^28^.

α_1_-AR are RCPGq proteins that are divided into α_1A_, α_1B_ and α_1D_ subtypes. The binding of α_1_-AR antagonists to these receptors counteracts Gq-activated InositolTrisPhosphate/calcium/Myosin Light Chain Kinase pathway and leads to relaxation of smooth muscles, making α_1_-AR antagonists efficient drugs to treat lower urinary tract symptoms in patients with benign prostatic hyperplasia (BPH). Naftopidil has been commercialized in Japan for this therapeutic indication and surprisingly, observational cohort studies reported that the incidence of prostate cancer was significantly lowered in men who received Naftopidil as compared with that of another α_1A_-AR antagonist as Tamsulosin^29^. Whereupon, Naftopidil was described to induce apoptosis in vitro in mesothelioma^30^ and to inhibit growth of several cancer cells in vitro and in vivo^31–33^. Moreover, it efficiently sensitized PC-3 xenograft cells to radiation and docetaxel-induced apoptosis^34,35^. Study of molecular mechanisms involved in apoptosis events highlighted that its action could be independent of α_1_-AR blocking^30,36^ and it was reported that Naftopidil bound directly to tubulin and inhibited its polymerization^37^. Its anticancer activity led to the development of more potent Naftopidil derivatives whose lead molecule, HUHS1015, was reported to transcriptionally increase Noxa and Puma expression^38^. This effect on BH3-only proteins could be used relevantly to sensitize ovarian carcinoma cells to innovative therapeutics and to justify Naftopidil repurposing in ovarian cancer management.

Based on these arguments, the aim of this study was to determine whether Naftopidil exerted an anticancer activity on ovarian cancer cells and whether it increased the [BH3-only]/[antiapoptotic] protein ratio and thus sensitized these cells to innovative treatments.

## Results

### Naftopidil inhibits proliferation of ovarian cancer cells

**Fig. 1:**
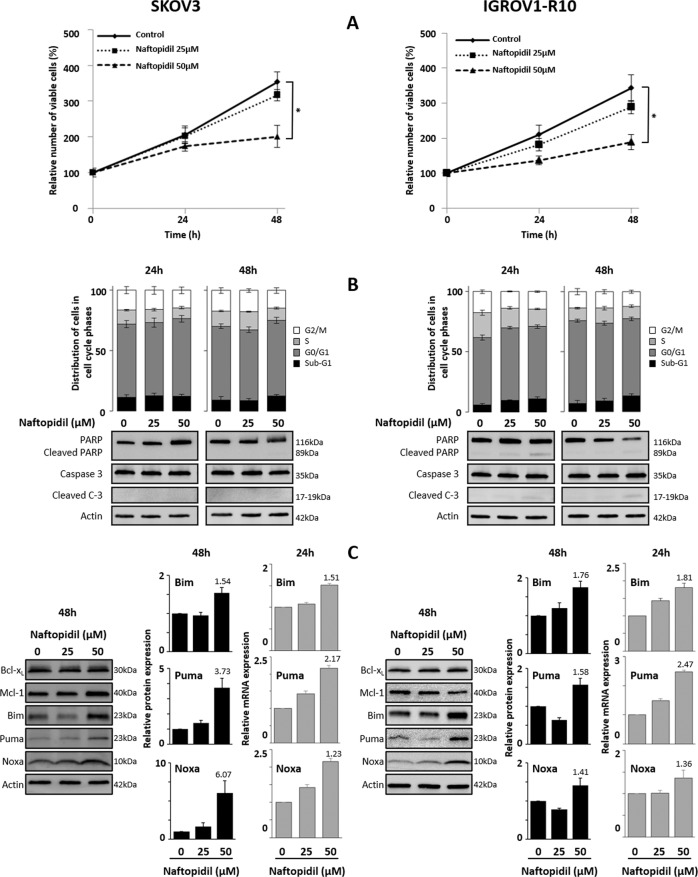
Naftopidil has a dose-dependent antiproliferative effect on ovarian cell lines and increases the expression of BH3-only proteins. Effects of increasing concentrations of Naftopidil (0, 25, 50 µM) were investigated in SKOV3 (left column) and IGROV1-R10 (right column) ovarian cancer cell lines after 24- and 48-h exposures **a** on cellular proliferation by the Trypan blue exclusion test (curves represent the relative viable cells number in the treated cells normalized to that in T0 condition), and **b** on apoptosis by analyzing the distribution of cells in the cell cycle phases using flow cytometry (upper panel) and PARP and Caspase 3 cleavages using western blot (lower panel). **c** After a 48-h treatment, effect on the Bcl-2 family members was studied using western blot analysis (left panel), expression levels of BH3-only proteins were quantified using ImageJ software and normalized to that of actin (histograms represent the relative expression of treated conditions normalized to that of control conditions) (middle panel); and after a 24-h exposure on BH3-only gene transcription by studying mRNA expression levels by RT-qPCR (histograms represent the relative mRNA expression of treated conditions normalized to that of control conditions) (right panel) (*N* = 3). Results were considered statistically different if *p < 0.05, **p < 0.01, ***p < 0.001.

Ovarian cancer cell lines SKOV3 and IGROV1-R10 were exposed to increasing concentrations of Naftopidil for 24 and 48 h. Whereas 25 µM Naftopidil had only a marginal antiproliferative action, a 48-h treatment with 50 µM Naftopidil significantly reduced growth on both cell lines suggesting that Naftopidil exerted a dose-dependent cytostatic effect (Fig. 1a). In these conditions, Naftopidil neither exerted any blockade in a specific phase of the cell cycle, nor induced any cytotoxic effects, as observed by the absence of cellular detachment (Supplementary Fig. S1) and the lack of accumulation of sub-G1 events (Fig. 1b). The absence of PARP and Caspase 3 cleavage also confirmed that Naftopidil did not trigger apoptotic cell death in these models (Fig. 1b). Taken together these results elicit that, in the tested conditions, Naftopidil exerts a cytostatic effect on ovarian cancer cell lines.

### Naftopidil increases the expression of the proapoptotic Bim, Puma and Noxa proteins in a dose- and transcriptional-dependent manner

We then studied the effect of Naftopidil on the expression of Bcl-2 family members. Whereas Naftopidil did not greatly modulate the expression of the antiapoptotic Bcl-x_L_ and Mcl-1 proteins after 48 h of treatment, it increased that of the proapoptotic BH3-only Bim, Puma and Noxa proteins as depicted by western blot and densitometry quantification (Fig. 1c). Furthermore, a 24-h treatment increased Bim, Puma and Noxa mRNA expression in a dose-dependent manner, suggesting that Naftopidil increases these proapoptotic BH3-only proteins through transcription-dependent mechanisms (Fig. 1c).

### Naftopidil increases the expression of Bim, Puma and Noxa in an α_1_-AR-independent way

**Fig. 2:**
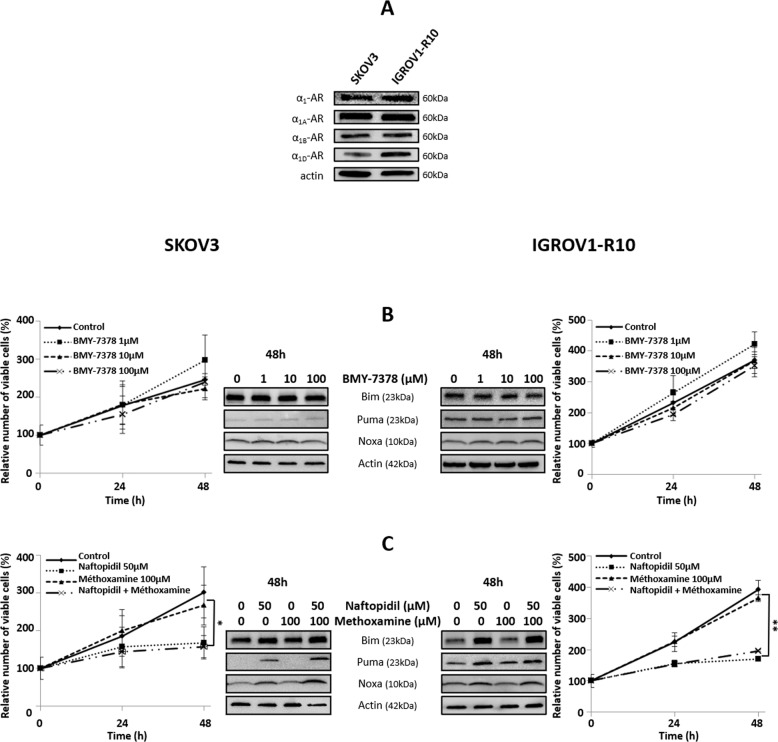
α_1D_-AR pathway is not involved in Naftopidil-induced BH3-only proteins. **a** Expression of α_1_, α_1A_, α_1B_ and α_1D_-AR proteins was assessed by western blot for SKOV3 and IGROV1-R10 cell lines. The effect of increasing concentrations of BMY-7378 (0, 10, 100 µM) on SKOV3 (left column) and IGROV1-R10 (right column) cells was analyzed (**b**) after a 24- and 48-h treatment on proliferation by the Trypan blue exclusion test (curves represent the relative viable cells number in the treated cells normalized to that in T0 condition) (left and right panels) and after a 48-h treatment on the BH3-only members expression by western blot (middle panel). The impact of the Methoxamine (100 µM) and Naftopidil (50 µM) combination was analyzed (**c**) after a 24- and 48-h treatment on proliferation by the Trypan blue exclusion test (curves represent the relative viable cells number in the treated cells normalized to that in T0 condition) (left and right panels) and after a 48-h treatment on the BH3-only member expression by western blot (middle panel) (*N* = 3). Results were considered statistically different if *p < 0.05, **p < 0.01, ***p < 0.001.

As Naftopidil is an α_1_-AR antagonist, we wondered if its effects were mediated by these receptors. Our first result showed that both cell lines expressed the three isoforms of α_1_-AR (Fig. 2a). Then, we analyzed the effect of an analog of Naftopidil, the BMY-7378, that also antagonizes α_1A_- and α_1D_-AR. Results showed that, unlike to Naftopidil, BMY-7378 neither exerted any antiproliferative effect nor induced BH3-only protein expression (Fig. 2b), suggesting that α_1_-AR were not involved in Naftopidil effects. To confirm this result, we tested if the α_1_-AR agonist Methoxamine could counteract Naftopidil effects. As depicted, Methoxamine did neither prevent growth arrest nor Naftopidil-induced Bim, Puma and Noxa protein expression (Fig. 2c). Collectively these results imply that α_1_-AR pathway does not play a major role in Naftopidil effects.

### Naftopidil increases BH3-only protein expression through ER stress-induced ATF4 in SKOV3 cell line and JNK activation in IGROV1-R10 cell line

We then tried to decipher the molecular pathways involved in Naftopidil-increased BH3-only protein expression. Akt/mTOR pathway is known to repress BH3-only proteins through the FoxO3A transcription factor phosphorylation^39–41^ and Naftopidil was described to inhibit Akt phosphorylation in gastric and prostate cancer cells^32,34,42^. However, in our models Naftopidil did neither greatly modulate activation of Akt/mTOR/4E-BP1/P70S6K pathway nor decrease FoxO3A phosphorylation (Supplementary Fig. S2a). BH3-only proteins could also be regulated by p53 ^17,19,43^. However, SKOV3 cell line does not express this protein. Moreover, a Naftopidil treatment had no effect on p53 expression in IGROV1-R10 cell line and p53 knockdown did not counteract Naftopidil-increased BH3-only protein expression ruling out p53 contribution in the effects of Naftopidil (Supplementary Fig. S2b). These BH3-only proteins could also be induced by NFκB activation^17,21,22^ and ROS^24–26^. Nevertheless, the NFκB inhibitor, BAY11-7085, or the oxidative stress inhibitor, N-AcetylCysteine (NAC), were ineffective to repress BH3-only protein induction in the presence of Naftopidil, suggesting that these pathways are not involved (Supplementary Fig. S2c, d).

**Fig. 3:**
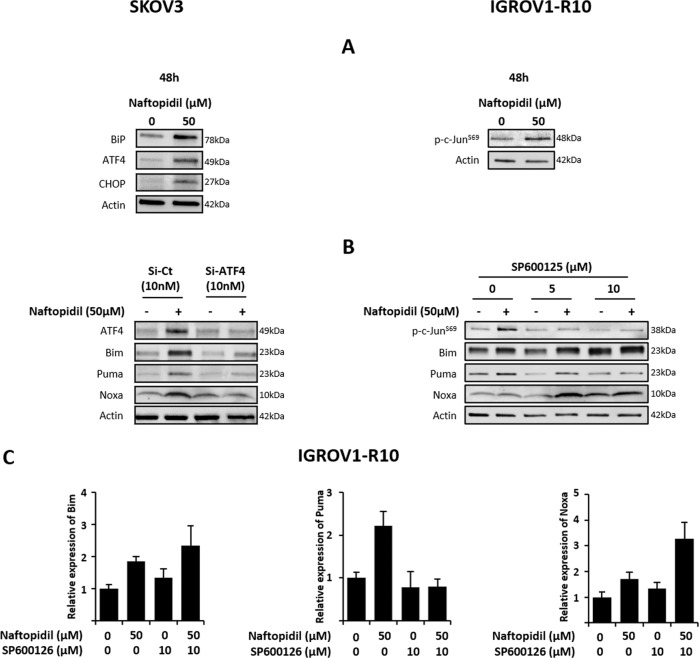
Naftopidil increases BH3-only protein expression through ER stress-induced ATF4 transcription factor in SKOV3 cells and through JNK pathway in IGROV1-R10 cell line. **a** SKOV3 (left column) and IGROV1-R10 (right column) cells were treated with Naftopidil (50 µM) during 48 h and the effect on ER stress markers and on c-Jun phosphorylation was then assessed by western blot, respectively. **b** SKOV3 cells (left column) were transfected with control (Si-Ct) or ATF4 (Si-ATF4) siRNAs (10 nM) 24 h before a Naftopidil (50 µM) treatment for 48 h. Target protein and BH3-only member expression were then analyzed by western blot. IGROV1-R10 cells (right panel) were treated with increasing concentrations of SP600125 (0, 5, 10 µM) during 1 h 30 min before a Naftopidil (50 µM)/SP600125 (5 or 10 µM) combination 48-h treatment, target protein phosphorylation and BH3-only member expression were then analyzed by western blot and **c** expression levels were quantified using ImageJ software and normalized to that of actin for the Naftopidil/SP600125 10 µM combination (histograms represent the relative expression of treated conditions normalized to that of control conditions) (*N* = 3).

Naftopidil was described to bind tubulin and inhibit its polymerization^37^. Its ability to act as a microtubule-targeting agent (MTA) could explain its antiproliferative effect and its capacity to upregulate proapoptotic members of Bcl-2 family. Actually, MTA are known to increase BH3-only protein expression or activity through different molecular mechanisms, the best known of which are the JNK pathway^44^ and ER stress activation^45^. Results showed that whereas Naftopidil did not modulate the expression of ER stress markers, BiP, CHOP and ATF4, in IGROV1-R10 cell line (Supplementary Fig. S3), it strongly induced them in SKOV3 cells implying that Naftopidil triggered an ER stress in this model (Fig. 3a, left panel). Moreover, the use of siRNA-targeting ATF4 strongly decreased Naftopidil-induced ATF4 and BH3-only proteins suggesting that Naftopidil increased BH3-only protein expression mainly through this pathway in SKOV3 cells (Fig. 3b, left panel). As for IGROV1-R10, we showed that exposure to Naftopidil increased c-Jun phosphorylation (Fig. 3a, right panel) and that the JNK inhibitor, SP600125, prevented Naftopidil-induced Puma but had no effect on the Bim and Noxa upregulation, as depicted by western blot and densitometry, implying that other molecular pathways should be involved (Fig. 3b, right panel, 3c and Supplementary Fig. 3b).

### Naftopidil sensitizes SKOV3 and IGROV1-R10 cell lines to ABT-737, a Bcl-x_L_ inhibitor

**Fig. 4:**
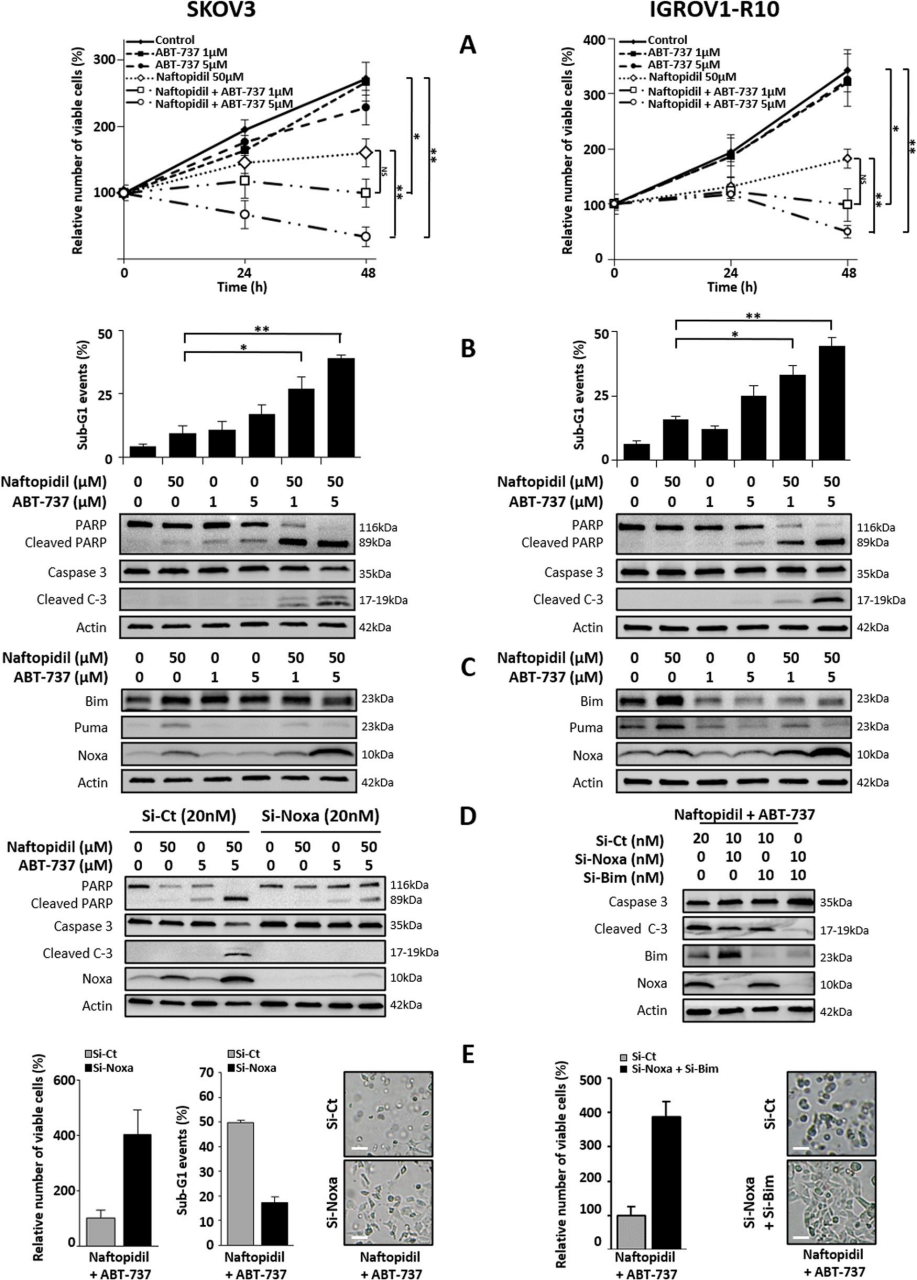
Naftopidil sensitizes ovarian cancer cells to ABT-737 through BH3-only protein-induced expression. Effects of Naftopidil (50 µM) and increasing concentrations of ABT-737 (0, 1, 5 µM) combination treatments were investigated in SKOV3 (left column) and IGROV1-R10 (right column) cell lines after 24 and 48 h **a** on proliferation by the Trypan blue exclusion test (curves represent the relative viable cells number in the treated cells normalized to that in T0 condition); and only after 48 h **b** on apoptosis by analyzing the rate of sub-G1 events using flow cytometry (upper panel) and PARP and caspase 3 cleavages by western blot (lower panel), and **c** on the expression of BH3-only proteins by western blot (the same western blot was used for actin protein expression in **b**, **c**). Twenty-four hours before Naftopidil/ABT-737 (5 µM) treatment, SKOV3 cells were transfected with control (Si-Ct) or Noxa (Si-Noxa) siRNAs (20 nM) while IGROV1-R10 cells were transfected with control (Si-Ct) or Noxa (Si-Noxa) and Bim (Si-Bim) siRNAs (20 nM), **d** after a 48-h combination treatment, target protein expression and PARP and Caspase 3 cleavages as markers of apoptosis were observed by western blot and **e** the number of viable cells was assessed by the Trypan blue exclusion test (histograms represent the relative viable cells number in treated cells transfected with Si-Noxa for SKOV3 cells or Si-Noxa and Si-Bim for IGROV1-R10 cells normalized to that in transfected with Si-Ct) (left panel), percentage of sub-G1 events was obtained by flow cytometry (histograms represent the percentage of sub-G1 events in treated cells transfected with Si-Noxa for SKOV3 cells normalized to that in transfected with Si-Ct) (middle panel) and the cell morphology of treated cells transfected with Si-Noxa for SKOV3 cells or Si-Noxa and Si-Bim for IGROV1-R10 cells was observed by optical microscopy (scale bar = 100 μm) (right panel) (*N* = 3). Results were considered statistically different if *p < 0.05, **p < 0.01, ***p < 0.001.

As Naftopidil increases the expression of BH3-only proteins, it could be an excellent candidate to counteract the activity of Mcl-1 and therefore to sensitize our models to ABT-737. To verify this hypothesis, cells were cotreated with Naftopidil and increasing concentrations of ABT-737. When used as a single agent, ABT-737 did not trigger any pronounced antiproliferative or cytotoxic effects whatever the concentration used (Fig. 4a). In contrast, its combination with Naftopidil provoked a massive apoptosis confirming the ABT-737-sensitizing effect of Naftopidil on both cell lines (Fig. 4a, b).

Interestingly, ABT-737 prevented the ability of Naftopidil to induce Puma expression in both cell lines and that of Bim in IGROV1-R10 cells. On the contrary, this BH3-mimetic triggered an overinduction of Noxa protein in our two models (Fig. 4c). Noxa silencing effect was then tested to evaluate the role of Noxa overexpression in Naftopidil/ABT-737 combination-mediated cell death. In SKOV3 cell line, Noxa knockdown was sufficient to protect against apoptosis as illustrated by the reduction of PARP and Caspase 3 cleavages (Fig. 4d), the increase of cell viability, the decrease of sub-G1 events and cell detachment (Fig. 4e, left panel). As for IGROV1-R10, Noxa silencing only weakly protected from cell death and led to an overexpression of Bim protein in this condition (Supplementary Fig. S4a–c). To test the functionality of this compensatory effect, Noxa and Bim were both silenced before exposing cells to the combined treatment which completely counteracted apoptosis induction (Fig. 4e, right panel). Collectively, these results suggest that Naftopidil-induced BH3-only protein sensitizes ovarian cancer cell lines to ABT-737.

### Naftopidil sensitizes SKOV3 and IGROV1-R10 cell lines to Trametinib, an MEK inhibitor

**Fig. 5:**
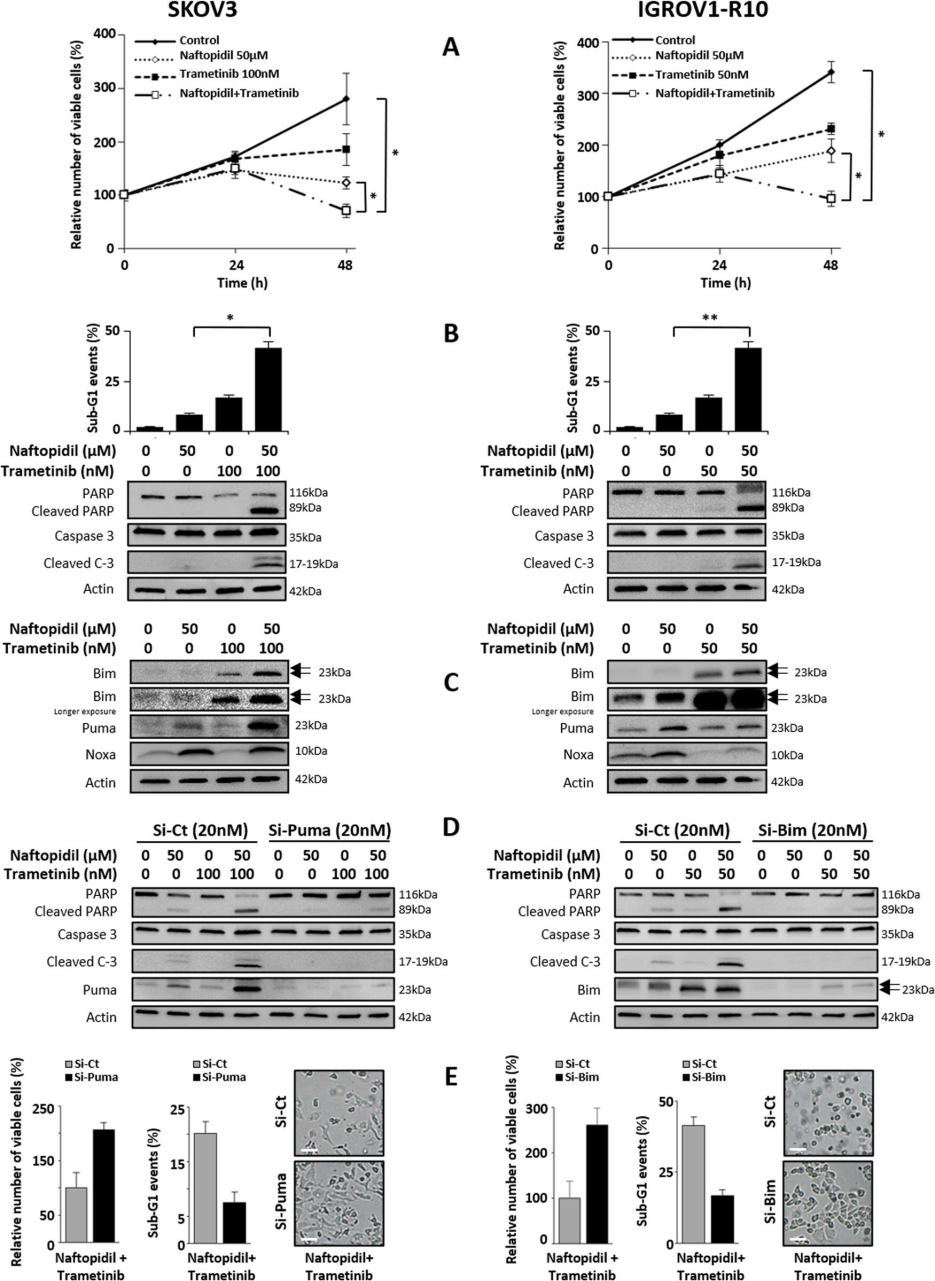
Naftopidil sensitizes ovarian cancer cells to Trametinib through BH3-only protein-induced expression. Effects of Naftopidil (50 µM) and Trametinib (100 nM for SKOV3 cells (left column) and 50 nM for IGROV1-R10 cells (right column)) combination treatment were investigated after 24 and 48 h **a** on proliferation by the Trypan blue exclusion test (curves represent the relative viable cells number in the treated cells normalized to that in T0 condition); and only after 48 h **b** on apoptosis by analyzing the rate of sub-G1 events using flow cytometry (upper panel) and PARP and caspase 3 cleavages by western blot (lower panel), and **c** on the expression of BH3-only proteins by western blot (the same western blot was used for actin protein expression in **b**, **c**). Twenty-four hours before Naftopidil/Trametinib treatment, SKOV3 cells were transfected with control (Si-Ct) or Puma (Si-Puma) siRNAs (20 nM) while IGROV1-R10 cells were transfected with control (Si-Ct) or Bim (Si-Bim) siRNAs (20 nM), **d** after a 48-h combination treatment, target proteins expression and PARP and Caspase 3 cleavages as markers of apoptosis were observed by western blot and **e** the number of viable cells was assessed by the Trypan blue exclusion test (histograms represent the relative viable cells number in treated cells transfected with Si-Puma for SKOV3 cells or Si-Bim for IGROV1-R10 cells normalized to that in transfected with Si-Ct) (left panel), percentage of sub-G1 events was obtained by flow cytometry (histograms represent the percentage of sub-G1 events in treated cells transfected with Si-Puma for SKOV3 cells or Si-Bim for IGROV1-R10 cells normalized to that in transfected with Si-Ct) (middle panel) and the cell morphology of treated cells transfected with Si-Puma for SKOV3 cells or Si-Bim for IGROV1-R10 cells was observed by optical microscopy (scale bar = 100 μm) (right panel) (*N* = 3). Results were considered statistically different if *p < 0.05, **p < 0.01, ***p < 0.001.

It was already demonstrated that the MEK inhibitor Trametinib, through its ability to prevent ERK phosphorylation, led to an increase of the dephosphorylated active form of Bim in SKOV3 and IGROV1-R10 cells and to the induction of Puma in IGROV1-R10 cell line^12^. This imbalance creates a favorable state that could sensitize ovarian cancer cells to innovative treatments as Naftopidil. To address this purpose, cell lines were cotreated with Naftopidil and Trametinib. As for Naftopidil, Trametinib treatment only slowed down cell growth in both cell lines (Fig. 5a), whereas the combination of these two molecules elicited a massive apoptosis (Fig. 5a, b). It should be noticed that this combination did not exert a strong cytotoxic effect on the non malignant ovarian T1074 cell line (Supplementary Fig. S5). However, it should be remarked that the Naftopidil/Trametinib combination was less efficient than the Naftopidil/ABT-737 one on both cell lines, as depicted by the difference between caspase3-7-positive cells/confluency percentages (Supplementary Fig. S6).

As expected, Trametinib increased the dephosphorylated active form of Bim expression in both cell lines. Trametinib also induced Puma expression in IGROV1-R10 cells and enhanced the Naftopidil-induced Puma expression in SKOV3 cells. Finally, Trametinib treatment had no impact on the expression of Noxa in SKOV3 cells but downregulated it in IGROV1-R10 cells (Fig. 5c). Based on these results, we investigated the role of Bim and Puma overexpression in cell death mediated by Naftopidil/Trametinib combination and silenced each BH3-only member before exposing cell lines to cotreatments. Whereas silencing Bim had no effect in SKOV3 cells (Supplementary Fig. S7a, b, right panel), Puma knockdown strongly abrogated apoptosis induced by the Naftopidil/Trametinib combination (Fig. 5d, e, left panel). As for IGROV1-R10, silencing Puma did not protect from the apoptosis triggered by the cotreatment (Supplementary Fig. S7a, b, left panel). In contrast, Bim silencing completely abrogated cell death mediated by this combination (Fig. 5d, e, right panel). Altogether, these results highlight that Naftopidil, through the induction of BH3-only proteins, imbalances the [pro-]/[antiapoptotic] protein ratio and sensitizes ovarian cancer cell lines to Trametinib.

### Effects of Naftopidil combination with ABT-737 or Trametinib on HGSOC-derived organoids

**Fig. 6:**
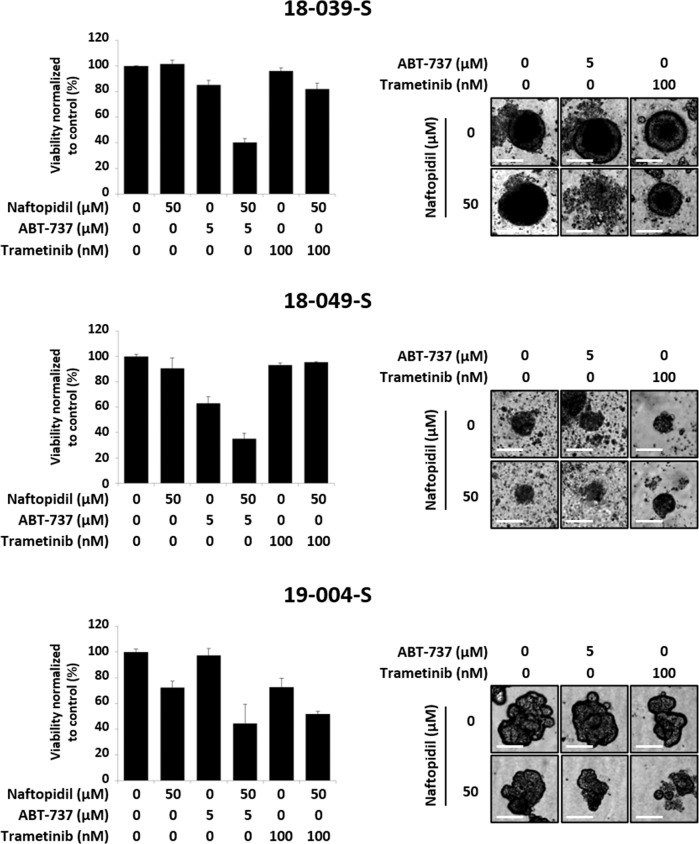
Naftopidil/ABT-737 and Naftopidil/Trametinib combinations trigger cell death in PDOs. Three PDOs were treated with Naftopidil (50 µM) and ABT-737 (5 µM) or Trametinib (100 nM) for 48 h. **a** Viability of PDOs was measured in each condition (histograms represent the percentage of viability in treated conditions normalized to that in control condition). **b** The effect of these combinations on PDOs morphology was assessed by optical microscopy (scale bar = 100 μm).

In order to appreciate the cytotoxic effect of Naftopidil/ABT-737 or Naftopidil/Trametinib combinations in a model closer to clinical situation, we tested these treatments on ovarian cancer organoids. Three organoids lines were established from ascites of patients with high-grade serous ovarian cancer (HGSOC) and the HGSOC histology was confirmed by a pathologist from the Cancer Center François Baclesse. These organoids were treated with Naftopidil in combination with ABT-737 or Trametinib. Viability assay showed a drastic decrease in cell viability in the Naftopidil/ABT-737 strategy. The cytotoxic effect of Naftopidil/Trametinib combination was also observed but results were more heterogeneous (Fig. 6). These results were confirmed by optical microscopy and revealed that although Naftopidil, ABT-737 or Trametinib used as single agents did not have noticeable effect on organoid morphological features, Naftopidil/ABT-737 combination disintegrated the structure of this 3D model (Fig. 6). Taken together, Naftopidil can sensitize patient-derived organoids (PDO) to ABT-737 and, to a lesser extent to Trametinib, opening new perspectives for ovarian cancer outcomes.

## Discussion

Our study showed that Naftopidil inhibited growth of ovarian cancer cells, which is consistent with results obtained with renal^31^ or prostate^32,33^ cancer cells for the same range of concentrations. However, in contrary to these models, this effect was not accompanied by G0/G1 cell cycle arrest. Naftopidil was able to induce p21 and p27 in ovarian cancer cell lines (data not shown). As these cyclin-kinase inhibitors are known to impede several check-points of the cell cycle, their upregulation could lead to a slow-down of the whole cell cycle instead of a blockade in a specific phase. To support this point, the Naftopidil derivative molecule Compound 13 was also described to slow down prostate cancer cell line growth without G0/G1 cell cycle arrest^46^. Naftopidil did not trigger apoptosis when used as a single agent and this result was supported by previous studies in PC-3 and LNCap cells^32,33^. This effect seemed however to be dependent on cellular context as Naftopidil was described to trigger cell death in other models such as bladder cancer^36^ or mesothelioma cell lines^30,47^. Nevertheless, the concentrations used in mesothelioma models were twice much higher which could explain this discrepancy.

The investigation of Naftopidil effects on Bcl-2 family members in our models revealed that this compound transcriptionally induced the BH3-only proteins Bim, Puma and Noxa. Transcriptional induction of Noxa and Puma were previously observed with the Naftopidil analog HUHS1015 in mesothelioma cell lines^38^. It could be suggested that the induction of these BH3-only proteins was not efficient enough to counteract antiapoptotic activity which could explain the absence of apoptosis induction upon Naftopidil treatment in ovarian carcinoma models.

The use of another specific α_1_-AR antagonist, BMY-7378, did neither mimic Naftopidil antiproliferative effect nor its ability to induce BH3-only. Besides, the α_1_-AR agonist Methoxamine did not counteract these effects. This suggested that α_1D_-AR should not be involved in Naftopidil effects. This result is supported by other observations that highlighted that anticancer α_1_-AR antagonist activity is largely independent of α_1_-AR blockade^28,30,33,36^. It is noteworthy that the discrepancy between BMY-7378 and Naftopidil antiproliferative effects was also observed in prostate cancer^33^.

Naftopidil was reported to bind α- and β-tubulin, and, to inhibit their polymerization^37^, and it has been widely reviewed that disturbing microtubules organization led to a stress signal that resulted in the triggering of molecular transduction pathways including ER stress, JNK activation and subsequent upregulation and increased activity of proapoptotic members^18,44,48–52^. In our study, Naftopidil actually activated ER stress and ATF4 transcription factor which led to BH3-only protein induction in SKOV3, but not in IGROV1-R10 cell line, supporting that this effect is cell-context dependent. Besides, it is noteworthy that ER stress is already strongly activated in basal conditions in IGROV1-R10 cell line and that Naftopidil could not overinduce this phenomenon. As for this latter cell line, our results highlighted that Naftopidil induced Puma through JNK/c-Jun pathway. However, Bim and Noxa induction were not counteracted by JNK inhibition and further work is required to identify the molecular mechanism. Collectively, these results show for the first time that Naftopidil induced ER stress and JNK activation and that, depending on cellular context, these molecular pathways are involved in BH3-only protein increase in ovarian carcinoma.

Naftopidil increased the [BH3-only]/[antiapoptotic] protein ratio and sensitized ovarian carcinoma cells to ABT-737. Analysis of ABT-737 effects on BH3-only proteins revealed that it downregulated both basal and Naftopidil-induced Puma expression, a result that was also observed in colon and myeloma cell lines^53,54^. The effect of ABT-737 on Bim expression seemed to be cellular context dependent as it led to an increase of Bim in SKOV3 cells and to a downregulation of basal and Naftopidil-induced Bim in IGROV1-R10 cells which is in agreement with our previous work^55^. On the other hand, ABT-737 induced Noxa and upregulated Naftopidil-induced Noxa expression. The protective effect of Noxa knockdown in SKOV3 cells suggests that this overinduction is the cause of the apoptosis induction and consequently that the upregulation of Noxa induced by Naftopidil/ABT-737 combination was strong enough to efficiently counteract Mcl-1 activity. This result is in complete agreement with the crucial role played by Noxa/Mcl-1 axis in ABT-737 resistance in several solid cancer localizations^56,57^. It is noteworthy that in IGROV1-R10, inhibiting Noxa in the cotreatment condition led to an upregulation of Bim that abrogated the protective effect of siRNA-targeting Noxa. This effect was also observed in HeLa cells but the molecular mechanism was not deciphered^58^. This suggests a compensatory relationship between Noxa and Bim and it could be addressed that Bim and Noxa act together to efficiently sensitize IGROV1-R10 cells to the Naftopidil/ABT-737 combination.

It should also be remarked that combining MTAs with BH3-mimetics to imbalance Bcl-2 family ratio is a strategy that has been proven very efficient in many solid cancers treatments in vitro and in patient-derived xenograft model^59,60^. This combination offers interesting perspectives for the clinical use of ABT-263 (Navitoclax, the orally available ABT-737 analog). Due to its ability to antagonize the survival function of Bcl-x_L_ in platelets^61^, Navitoclax triggers thrombocytopenia, which is its major dose-limiting side-effect. As Naftopidil synergizes with ABT-737, it might allow a dose reduction of ABT-263, thereby alleviating this BH3-mimetic side-effect.

Trametinib is a Food and Drug Administration- and European Medicines Agency-approved MEK 1/2 inhibitor used to treat metastatic melanoma with B-Raf mutations and it entered phase II/III clinical trials in monotherapy for low-grade serous ovarian cancers (NCT02101788). Trametinib, through MEK deactivation, impeded Bim phosphorylation on serine 69^12^, which, as a consequence, inhibited its targeting of the proteasome and increased the Bim active form^62^. Trametinib and another MEK inhibitor were also found to increase Puma expression in cancer cells^12,63^. We then investigated if the Naftopidil/Trametinib combination could induce BH3-only proteins efficiently enough to overcome antiapoptotic activity. As expected, Trametinib induced dephosphorylated Bim active form and slightly induced Puma expression in both cell lines but this effect was not strong enough to trigger apoptosis. However, Trametinib strengthened Naftopidil-induced Bim and Puma which was efficient enough to buffer antiapoptotic activities. The protective effect of siRNA-targeting Puma in SKOV3 cells suggested that Puma played a crucial role in Naftopidil/Trametinib combination-induced apoptosis whereas Bim seemed to be the major inductor of apoptosis in IGROV1-R10 cell line. It should be noticed that SKOV3 cell line expressed Bim to a lesser extent than IGROV1-R10 cells which could explain why Bim had a modest role in Naftopidil/Trametinib combination-triggered apoptosis in this cell line. These results also highlight that ovarian cancer cell death could be achieved without directly inhibiting Mcl-1 and/or Bcl-x_L_, which opens new therapeutic perspectives for the management of ovarian cancer. Nevertheless, the comparison of the cytotoxic effect of the two combinations suggested that Naftopidil/ABT-737 was more potent that Naftopidil/Trametinib in both cell lines and this conclusion was also observed for PDO experiments.

Tumor organoids represent relevant 3D ex vivo models that mimic the tumor from which they are derived. They are obtained from culture of tumor cells embedded in basement membrane matrix in presence of a cocktail of growth factors and molecules to recreate cell niche and allow long-term growth. Emerging evidences indicate that tumor organoids recapitulate patient response in the clinic suggesting they could be used to enable precision medicine^64,65^. Ovarian cancer organoids have recently been successfully established and closely recapitulate the genetic and morphological heterogeneous composition of cancer cells in the original tumor^66,67^. Ascites-derived HGSOC organoids treated with Naftopidil/ABT-737 combination appeared disintegrated whereas Naftopidil/Trametinib treatment seemed less cytotoxic. This result associated to in vitro responses support that simultaneously upregulating BH3-only proteins and directly inhibiting Bcl-x_L_ through BH3-mimetics is a more efficient strategy than overinducing BH3-only through molecular pathways that could be subjected to mutations or compensatory mechanisms resulting from complex crosstalks and feedback loops. In this context, resistance to MEK inhibitors has been extensively described and it is quite possible that the PDO used in our study could harbor mutations in several transduction pathways or enhanced expression of receptor tyrosine kinases that could explain Trametinib loss of activity^68^. Finally, it was recently demonstrated that inflammatory micro-environment could account for Trametinib resistance in PDO cultures from KRAS-mutant colorectal tumors and increased expression of this panel of inflammatory genes was significantly correlated with colorectal cancer overall survival in TCGA database^69^. Proinflammatory environment might also hamper Trametinib activity in our model and further investigations should be undertaken to underpin this hypothesis.

Collectively, these results show that Naftopidil has an antiproliferative effect on ovarian cancer cell line. Through its capacity to inhibit cancer cell growth when used as a single agent, Naftopidil could be considered as a cytostatic drug which could open new perspectives to use this compound. Actually, the goal of cytostatic drugs is to slow proliferation and generate a lap of disease stabilization that will delay the reintroduction of cytotoxic drugs and by this way will achieve new end points of clinical efficacy such as time of progression, quality of life and survival^70^. Even if Naftopidil has no indication for ovarian cancer yet, its antiproliferative action could allow this compound to be suggested as a maintenance treatment for spacing out recurrence episodes. Moreover, whatever the molecular pathway implied, Naftopidil is able to induce the BH3-only Bim, Noxa and Puma in ovarian carcinoma which sensitizes to anti-Bcl-x_L_ strategies or to Trametinib (Supplementary Fig. S8). Our HGSOC PDO models also suggest that these combinations could have relevance in clinic. Despite its short half-life^71^, Naftopidil is used per os for BPH treatment suggesting that it reaches genital tractus and is effective via oral administration. This route of administration could be easier to use especially if patients have to take Naftopidil for long course. As Naftopidil is already commercialized and seems to have a good tolerance, this work could open new indications for this drug and enlarge the field of innovative strategies for ovarian cancer management.

## Material and methods

### Cell culture and treatment

The human platinum-resistant ovarian carcinoma cell lines SKOV3 and IGROV1-R10 were used. SKOV3 cell line was obtained from the American Type Culture Collection. IGROV1-R10 cell line was established as described previously^72^ from the IGROV1 cell line, kindly provided by Dr. Jean Bénard (Institut Gustave Roussy, Paris, France). The cell lines were authenticated in April 2016 by Microsynth who compared their STR profiles with the ATCC database. They were grown in RPMI1640 (Gibco) medium supplemented with 2 mM Glutamax™, 25 mM HEPES (4-(2-hydroxyethyl)-1-piperazineethanesulfonic acid), 10% decomplemented FBS (Fetal Bovine Serum) (Gibco) and 33 mM sodium bicarbonate (Gibco) and were maintained in a 5% CO_2_ humidified atmosphere at 37 °C. Ovarian cancer cell lines were certified mycoplasma-free thank to a MycoAlert test.

Naftopidil, BMY-7378, BAY-11-7085 and SP600125 were supplied by Tocris (R&D Systems), ABT-737 and Trametinib by Selleckem, Methoxamine and NAC by Sigma-Aldrich. These compounds were stored as stock solutions in DMSO (Dimethyl sulfoxide) or in ultra-pure water at −80 °C. Controlled conditions were realized with DMSO or ultra-pure water vehicle, its percentage not exceeding 0.2% v/v. A total of 3 × 10^5^ SKOV3 cells and 5 × 10^5^ IGROV1-R10 cells were plated in 25-cm^2^ flasks 24 h before being treated. A total of 1.5 × 10^5^ SKOV3 cells and 3.5 × 10^5^ IGROV1-R10 cells were plated in 25-cm^2^ flasks 24 h before being transfected.

### SiRNAs transfection

Bim siRNA, denoted Si-Bim (siRNA antisense sequence: 5′-uaacagucguaagauaacctt-3′) and Puma siRNA, denoted Si-Puma (siRNA antisense sequence: 5′-uauacaguaucuuacaggctt-3′) were chemically synthesized by Eurogentec; Noxa siRNA, denoted Si-Noxa (SMARTpool), ATF4 siRNA, denoted Si-ATF4 (ON-TARGETplus SMARTpool), p53 siRNA, denoted Si-p53 (ON-TARGETplus SMARTpool), Si-GENOME nontargeting siRNA Pool#1, denoted Si-Control (SMARTpool), and ON-TARGETplus Non-Targeting siRNA Pool, denoted Si-Control (SMARTpool), were purchased from Dharmacon (Horizon Discovery). All siRNAs were received as annealed oligonucleotides. Briefly, the transfecting INTERFERin reagent (Polyplus Transfection) was added to siRNAs diluted in Opti-MEM Reduced Serum Medium (Gibco). Complexes were allowed to form for 10 min at room temperature before application to cells to reach a final concentration of 10 or 20 nmol/L. Control conditions were made with Si-GENOME nontargeting siRNA Pool#1 for Si-Bim, Si-Puma and Si-Noxa and with ON-TARGETplus Non-Targeting siRNA Pool for Si-ATF4 and Si-p53. Cells were treated according to the protocol described above 24 h after transfection.

### Proliferation analysis

Cell number and viability were estimated using the Trypan blue exclusion method.

### Cell cycle analysis by flow cytometry

Adherent and floating cells were pooled, washed with PBS (Phosphate-Buffered Saline) and fixed with ethanol 70%. Cells were then centrifuged at 2000 r.p.m. for 5 min and incubated for 30 min at 37 °C in PBS, to allow the release of low-molecular weight DNA. Cell pellets were stained with a solution composed by 200 µg/mL of RNAse A (Fisher Scientific) and 50 µg/mL of propidium iodide (Fisher Scientific) diluted in PBS. Samples were analyzed using Gallios flow cytometer (Beckman-Coulter) and cell cycle distribution and sub-G1 fraction were determined using Gallios software (Beckman-Coulter).

### Extraction of proteins and western blot analysis

Proteins were extracted as follows: cells were rinsed with ice-cold PBS and lysed in lysis buffer [15 mM HEPES, 50 mM KCl, 10 mM NaCl, 1 mM MgCl_2_, 0.25% glycerol, 0.5% *n*-Dodecyl-*β*-d-maltopyranoside (Affymetrix), 5 µM GuanosineDiPhosphate, 1 µM microcystin (Enzo Life Sciences), 1 mM Na_3_VO_4_, Complete Protease Inhibitor Cocktail (Sigma-Aldrich/Roche)] and incubated on ice for 30 min. After centrifugation at 10,000 r.p.m. during 10 min, proteins were quantified using the Bradford assay (Bio-Rad). Equal amounts of protein (20 µg) were separated by SDS-PAGE on a 4−15% gradient polyacrylamide Mini-PROTEAN® TGX™ precast gel (Bio-Rad) and transferred to PVDF membranes (Bio-Rad). Membranes were blocked 1 h at room temperature with 5% (v/v) nonfat dry milk in TBS (Tris-Buffered Saline) with 0.05% (v/v) Tween20 (T-TBS). Membranes were then incubated overnight at 4 °C with the following primary antibodies: anti-Mcl-1 (#5453), anti-Bcl-x_L_ (#2764), anti-Bim (#2819), anti-Puma (#12450), anti-Caspase 3 (#9662), anti-PARP (#9542), anti-Akt (#9272), anti-p-Akt^S473^ (#4060), anti-p-Akt^T308^ (#13038), anti-p-mTOR^S2448^ (#5536), anti-p-mTOR^S2481^ (#2974), anti-4E-BP1 (#9644), anti-p-4E-BP1^T70^ (#4370), anti-p70S6K (#9202), anti-p-p70S6K^T389^ (#9205), anti-FoxO3A (#2497), anti-p-FoxO3A^S253^ (#9466), anti-c-jun^S63^ (#2361), anti-p-SAPK/JNK^T183/Y185^ (#9251), anti-BiP (#3177), anti-ATF4 (#11815), anti-CHOP (#5554) purchased from Cell Signaling Technology; anti-Noxa (#114C307) purchased from Calbiochem; anti-α_1_-AR (#ab192614), anti-α_1A_-adrenergic receptor (#ab137123), anti-α_1B_-adrenergic receptor (#ab169523) purchased from Abcam; anti-α_1D_-adrenergic receptor (#AP20589a) purchased from Abgent; anti-p53 (DO-1) (#sc-126) purchased from Santa-Cruz; anti-Actin (#MAB1501) purchased from Merck Millipore. Membranes were then incubated 1 h at room temperature with the appropriate horseradish peroxidase-conjugate anti-rabbit (#7074, Cell Signaling Technology) or anti-mouse (#NA931V, Amersham) secondary antibodies. Signals were revealed using Clarity Werstern EleCtroLuminescence (ECL) detection reagent (Bio-Rad) and the ImageQuant® Las4000Series (GE Healthcare Life Sciences), and then quantified by pixel densitometry using the ImageJ® software. Western blots shown are from one experiment representative of at least three independent experiments and cell lysates.

### RNA extraction and real-time quantitative reverse transcription PCR (RT-qPCR)

Total RNAs were isolated from ovarian cancer cell lines using Trizol (Invitrogen, Life Technologies). RNA quantity and quality were assessed using the NanoDrop™ 2000 spectrophotometer (ThermoScientific). The first strand cDNA was synthesized using Omniscript reverse transcriptase kit (Qiagen) with random hexamers. cDNA (25 ng) was combined with 10 µmol/L of each forward and reverse primers, 50 µmol/L of the Taq-Man® probe and Taq-Man® Fast Universal PCR Master Mix (Applied Biosystems) in a 20 µL final reaction volume. Corresponding custom inventoried (ID: Bim HS00708019_s1, Puma HS00248075_m1, Noxa HS00560402_m1 and GAPDH HS99999905_m1) Taq-Man® Gene Expression Assays were used (Applied Biosystems). All PCR amplification reactions were carried out in triplicate and detection was done on an Applied ABI Prism 7500 Fast PCR System (Applied Biosystems). GAPDH was used as a housekeeping gene for normalization.

### Patient-derived organoids

#### Patient samples

Ascites fluid samples were collected from three patients with HGSOC at the Centre François Baclesse and sent to the laboratory promptly after drainage. The HGSOC histology was confirmed by a pathologist. Written informed consent was obtained for all subjects and the study was approved by the North West III’ ethical committee (IDRCB: 2018-A02152-53).

#### Organoid establishment and culture

Ascites were spun at 2000 r.p.m. to create cell pellets. Pellets were resuspended in “collecting medium” [RPMI1640 medium supplemented with 2 mM Glutamax™, 25 mM HEPES (Gibco), 10% decomplemented FBS (Gibco), 33 mM sodium bicarbonate (Gibco), 100 UI/mL of penicillin (Gibco), 100 µg/mL of streptomycin (Gibco) and 1% bovine serum albumin (Sigma-Aldrich)]. Tumor spheroids of 50–300 μm in diameter were selected using cell strainers (Endecotts) and enzymatically dissociated into single cells/small cell clusters using TrypLE Express (Gibco) for up to 10 min at 37 °C. The cell pellets were washed once with collecting medium and resuspended in a small volume of organoid culture medium [Advanced DMEM supplemented (Gibco) with 100 UI/mL of penicillin, 100 µg/mL of streptomycin, 1% GlutaMAX (Gibco), 1× B27 (Gibco), 1.25 mM NAC (Sigma-Aldrich), 50 ng/mL EGF (PeproTech), 20 ng/mL FGF-10 (PeproTech), 1 ng/mL FGF-2 (PeproTech), 500 nM A-83-01 (PeproTech), 10 µM Y27632 (Interchim), 1 µM SB202190 (PeproTech), 10 mM Nicotinamide (Sigma-Aldrich), 1 µM PGE2 (PeproTech), 100 µg/mL Primocin (InvivoGen), 50% Wnt3a, RSPO3, Noggin-conditioned media (L-WRN, ATCC), and 10% RSPO1-conditioned media (Cultrex HA-R-Spondin-1-Fc 293 T, Amsbio)] and mixed with a 1:1 volume of growth factor-reduced Matrigel (Corning). Drops of 50 µL of Matrigel/cell suspension (one drop and 10,000 cells per well) were distributed into a prewarmed 24-well plate (Eppendorf). Once the Matrigel was solidified, 500 μL of organoid culture medium was added to each well. The medium was changed twice a week and tumor organoids were passaged every 7−14 days by dissociation with TrypLE Express for up to 10 min at 37 °C. Single cells and small cell clusters were replated according to the procedure described above. Cryovials were prepared at regular intervals by dissociating and resuspending organoids in Recovery Cell Culture Freezing Medium (GIBCO) prior to be biobanked in liquid nitrogen.

#### Organoid viability assays

Organoid culture medium lacking *N*-Acetylcysteine, Y-27632 and primocin was mixed with a 1:1 volume of growth factor-reduced Matrigel and 40 µL of the solution was dispensed into white, clear bottom 96-well plates (Greiner). Tumor organoids were collected before being resuspended in 2% Matrigel/organoid culture medium lacking *N*-Acetylcysteine, Y-27632 and primocin and plating in 100 µL volume. The drugs (100 µL of volume) were added 1 h after plating the organoids. Forty-eight hours after adding the drugs, ATP levels were quantified using CellTiter-Glo 3D cell viability assay (Promega) according to the manufacturer’s instruction and luminescence was measured using Centro XS3 LB 960 (Berthold technologies) with Miko Win 2000 software. Results were normalized to vehicle.

### IncuCyte apoptosis analysis

SKOV3 and IGROV1-R10 cells were seeded at 3.5 × 10^3^ cells and 6 × 10^3^ per well respectively in 96-well plates in media. Cells were cultured at 37 °C and 5% CO_2_ and monitored using an IncuCyte® S3 (Essen BioScience). IncuCyte® Caspase-3/7 Green Apoptosis Assay Reagent (Essen BioScience) was added immediately following treatment and baseline images were taken using ×10 objective. The plate was scanned and fluorescent and phase-contrast images were acquired in real time every 1 h from one region per well. The caspase 3/7 reagent labels dead cells yielding green fluorescence. The live-cell phase contrast images were used to calculate confluence using the IncuCyte® software, and to provide morphology information. Accumulation of caspase 3/7 over time was normalized to confluence of cells.

### Statistical analysis

The values were presented as means ± SEM for at least three independent experiments. Mann−Whitney *U* test was used for statistical analysis. Results were considered statistically different if *p < 0.05, **p < 0.01, ***p < 0.001.

## Supplementary information

Supplementary Figure Legends

Supplementary data S1

Supplementary data S2

Supplementary data S3

Supplementary data S4

Supplementary data S5

Supplementary data S6

Supplementary data S7

Supplementary data S8
