# Supplementary material for: Bim, Puma and Noxa upregulation by Naftopidil sensitizes ovarian cancer to the BH3-mimetic ABT-737 and the MEK inhibitor Trametinib
Source: Cell Death Dis. 2020 May 18;11(5):380. doi: 10.1038/s41419-020-2588-8 (PMC7235085; doi:10.1038/s41419-020-2588-8)
Supplement: Supplementary file 1 — Supplementary Figure Legends [file 41419_2020_2588_MOESM1_ESM.docx]

**Supplementary Figures and legends**

**Supplementary data S1**

Morphological effects of Naftopidil on ovarian cancer cell lines. The effect of increasing concentrations of Naftopidil (0, 25, 50µM) on morphological features was assessed by optical microscopy (scale bar = 100μm) after 24 and 48-hour exposures. (N=3)

**Supplementary data S2**

The Akt/mTOR pathway, NFkB and ROS are not involved in Naftopidil-induced BH3-only proteins. Effects of a 48-hour treatment with increasing concentrations of Naftopidil (0, 25, 50µM) in SKOV3 cells (left panel) and IGROV1-R10 cells (right panel) were assessed **(A)** on Akt/mTOR pathway members by western blot **(B)** on p53 by western blot (upper panel) and on BH3-only protein expression in IGROV1-R10 cells transfected with control (Si-Ct) or p53 (Si-p53) siRNAs (20nM) 24 hours before treatment (lower panel). Effects on BH3-only protein expression were also analyzed by western blot on cells treated with **(C)** Naftopidil (50µM) and BAY11-7085 (5µM) combination during 48 hours or **(D)** Naftopidil (50µM) and increasing concentrations of NAC (pH adjusted to 7,3) (0, 5, 10µM) during 48 hours. (N=3)

**Supplementary data S3**

Naftopidil does not induce ER stress in IGROV1-R10 cell line and its combination with SP600125 inhibits Puma expression but does not reduce Naftopidil-induced Bim and Noxa up-regulation. **(A)** IGROV1-R10 cells were treated with Naftopidil (50µM) during 48 hours and the effect on ER stress markers was then assessed by western blot. **(B)** IGROV1-R10 cells were treated with different concentrations of SP600125 during 1h30 before being co-treated with a Naftopidil (50µM)/SP600125 combination for 48h, Bim, Puma and Noxa expressions were then analyzed by western blot. (N=3)

**Supplementary data S4**

Noxa silencing does not protect IGROV1-R10 cell line against Naftopidil/ABT-737 combination treatment. IGROV1-R10 cells were transfected with control (Si-Ct) or Noxa (Si-Noxa) siRNAs (20nM) 24 hours before a 48-hour treatment of Naftopidil (50µM) and ABT-737 (5µM). Effects on **(A)** the target protein expression and PARP and Caspase 3 cleavages used as markers of apoptosis were observed by western blot , **(B)** the number of viable cells was assessed by the Trypan blue exclusion test (histograms represent the relative viable cells number in treated cells transfected with Si-Noxa normalized to that in transfected with Si-Ct), percentage of sub-G1 events was obtained by flow cytometry (histograms represent the percentage of sub-G1 events in treated cells transfected with Si-Noxa normalized to that in transfected with Si-Ct) and the cell morphology of treated cells transfected Si-Noxa was observed by optical microscopy (x10) (right panel) ; and **(C)** impact on BH3-only protein expression was observed by western blot. (N=3)

**Supplementary data S5**

Naftopidil/Trametinib combination does not induce a strong cytotoxic effect on the non-malignant ovarian T1074 cell line. Effects of Naftopidil (50µM) and Trametinib (50nM or 100nM) combination treatment were investigated on T1074 cell line after 48 hours on **(A)** morphological features by optical microscopy (scale bar = 100 μm), **(B)** cellular proliferation by the Trypan blue exclusion test (histograms represent the relative viable cells number in the treated cells normalized to that in control condition), and on apoptosis by analyzing **(C)** the rate of sub-G1 events using flow cytometry and **(D)** PARP and Caspase 3 cleavages using western blot. (N=2)

**Supplementary data S6**

Naftopidil/ABT-737 combination is more cytotoxic than Naftopidil/Trametinib combination. SKOV3 (left panel) and IGROV1-R10 (right panel) cell lines were treated with Naftopidil (50µM) and **(A)** ABT-737 (5µM) or **(B)** Trametinib (100nM for SKOV3 and 50nM for IGROV1-R10 cells) during 48h and apoptosis was analyzed with the IncuCyte® Caspase-3/7 Green Apoptosis Assay. (N=3)

**Supplementary data S7**

Bim and Puma are not involved in the cytotoxic effect of the Naftopidil/Trametinib combination on SKOV3 and IGROV1-R10 cells, respectively. SKOV3 cells (left column) and IGROV1-R10 cells (right column) were, respectively, transfected with control or Bim (Si-Bim) siRNAs (20nM) (left panel) and control or Puma (Si-Puma) siRNAs (20nM) (right panel) 24 hours before a 48-hour Naftopidil (50µM)/Trametinib (100nM for SKOV3 and 50nM for IGROV1‑R10 cells) combination treatment. **(A)** The cell morphology was observed by optical microscopy (scale bar = 100μm), **(B)** the expression of target proteins and PARP and Caspase 3 cleavages used as markers of apoptosis were observed by western blot. (N=3)

**Supplementary data S8**

Molecular pathways involved in Naftopidil/ABT-737 and Naftopidil/Trametinib combinations in SKOV3 and IGROV1-R10 cell lines. **(A)** In SKOV3 cell line, Naftopidil induces Bim, Puma and Noxa transcription through ER stress/ATF4 pathway. The BH3-mimetic ABT-737 inhibits the activity of the anti-apoptotic Bcl-x_L_ and its combination with Naftopidil over-induces Noxa expression tipping the balance in favor of apoptosis. **(B)** The MEK inhibitor Trametinib induces the dephosphorylated active form of Bim and strengthens Naftopidil-induced Puma. Functional experiments demonstrated that Puma plays the key role in the induction of apoptosis probably because SKOV3 cell line expresses low basal level of Bim protein. **(C)** In IGROV1-R10, Naftopidil induces Bim, Puma and Noxa transcription through JNK/c-Jun molecular pathway at least for Puma protein expression. In the same way as for SKOV3 cell line, ABT-737 inhibits the activity of Bcl-x_L_ and reinforces Naftopidil-induced Noxa protein expression. However, inhibiting Noxa is not sufficient to protect IGROV1-R10 cell line from Naftopidil/ABT-737-induced apoptosis as knock-down of Noxa reinduces Bim protein that takes over to trigger cell death (the compensatory mechanism is not deciphered and is pointed by a red asterisk). As a consequence, the inhibition of both Noxa and Bim protein is required to thwart apoptosis. **(D)** Trametinib induces Puma and the dephosphorylated active form of Bim, which plays the major role in inducing apoptosis in Naftopidil/Trametinib combination. The lower basal level of Bim protein in SKOV3 compared to that in IGROV-R10 cell line is illustrated by a dashed line and a lighter shade of blue. The BH3-only proteins that play the key role in inducing apoptosis in combination treatments is indicated by a frame of blue light.
