# Supplementary figures and images for: Bim, Puma and Noxa upregulation by Naftopidil sensitizes ovarian cancer to the BH3-mimetic ABT-737 and the MEK inhibitor Trametinib
Source: Cell Death Dis. 2020 May 18;11(5):380. doi: 10.1038/s41419-020-2588-8 (PMC7235085; doi:10.1038/s41419-020-2588-8)

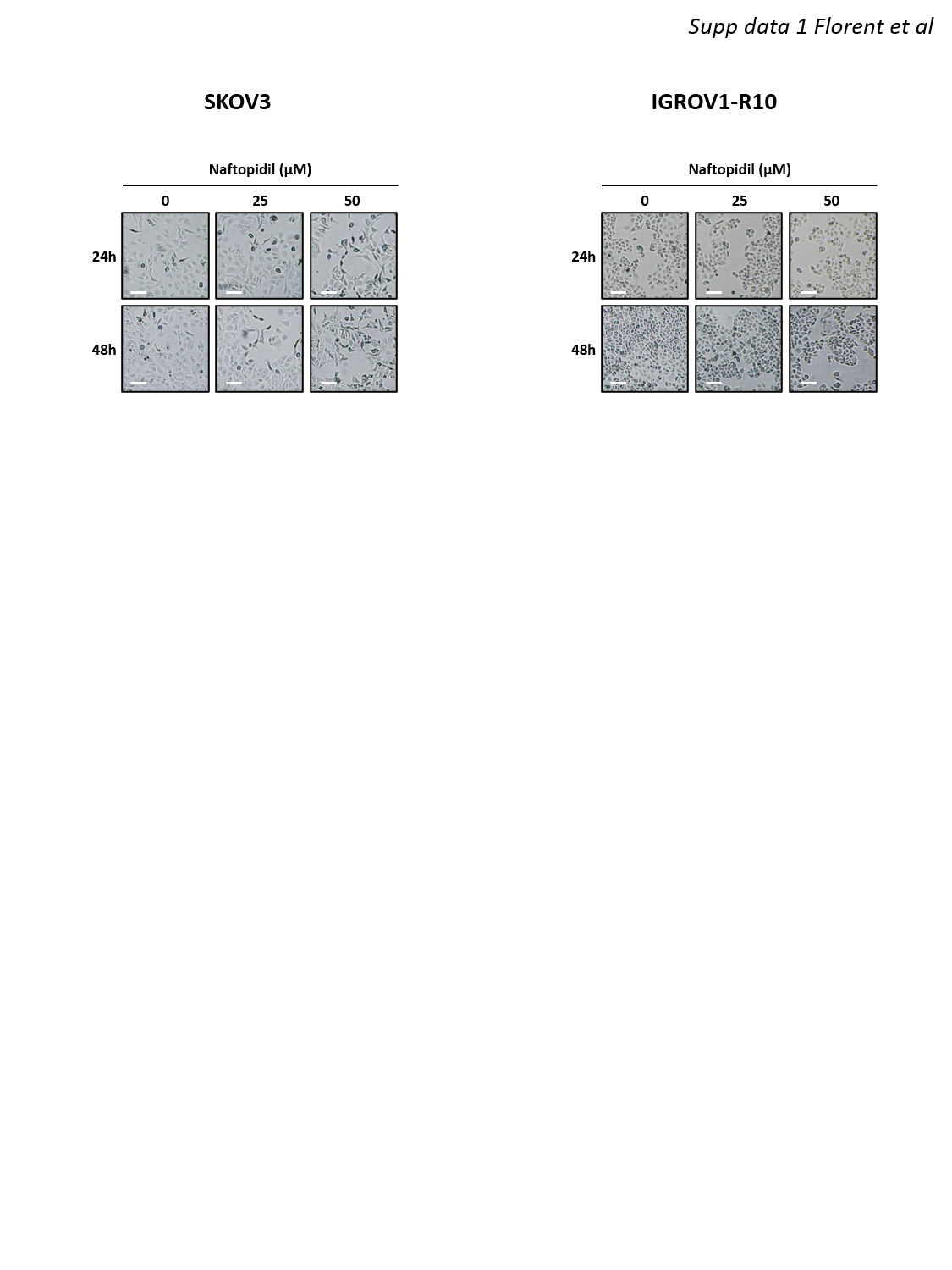

Supplement: Supplementary file 2 — Supplementary data S1 [file 41419_2020_2588_MOESM2_ESM.png]

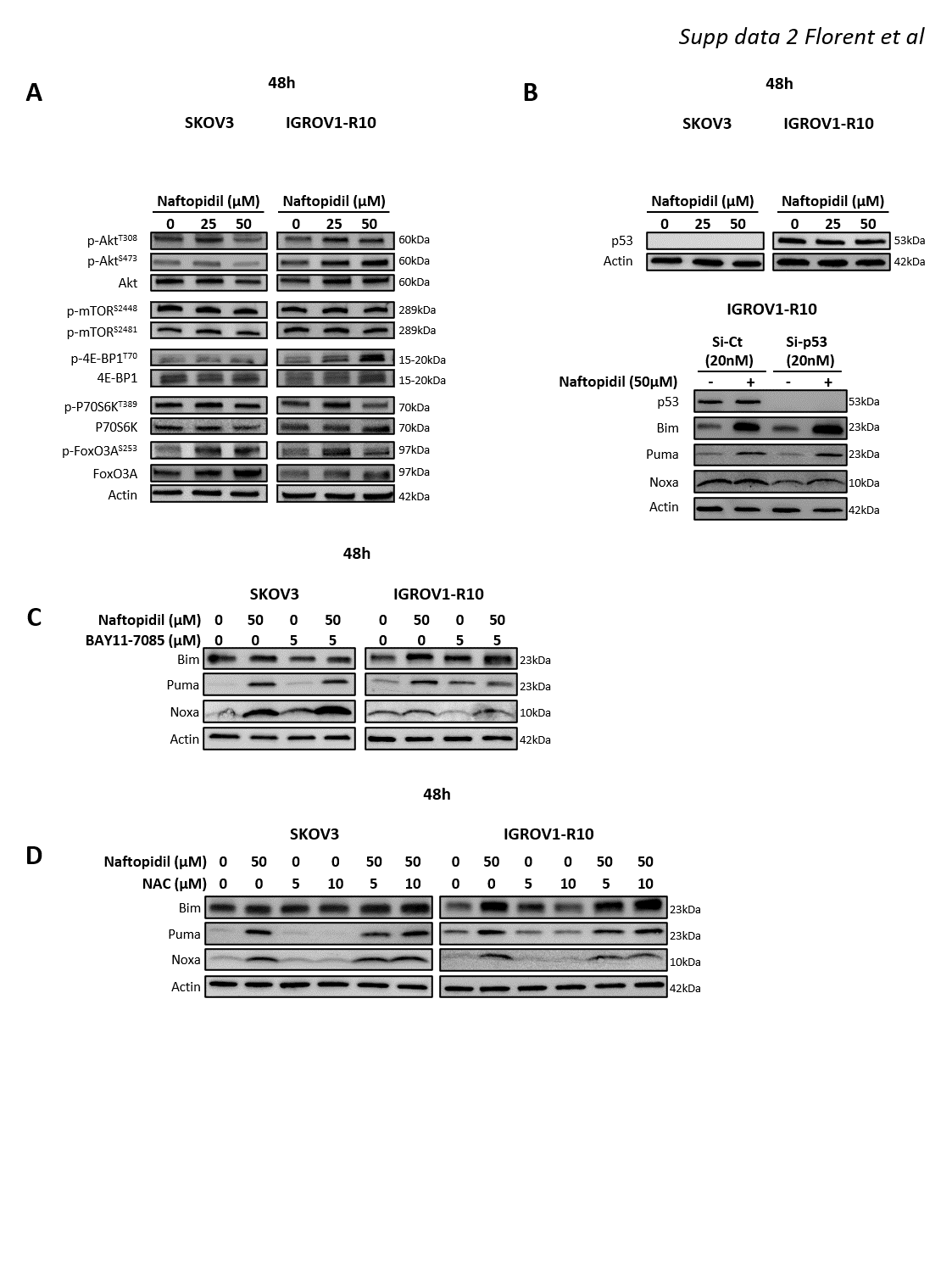

Supplement: Supplementary file 3 — Supplementary data S2 [file 41419_2020_2588_MOESM3_ESM.png]

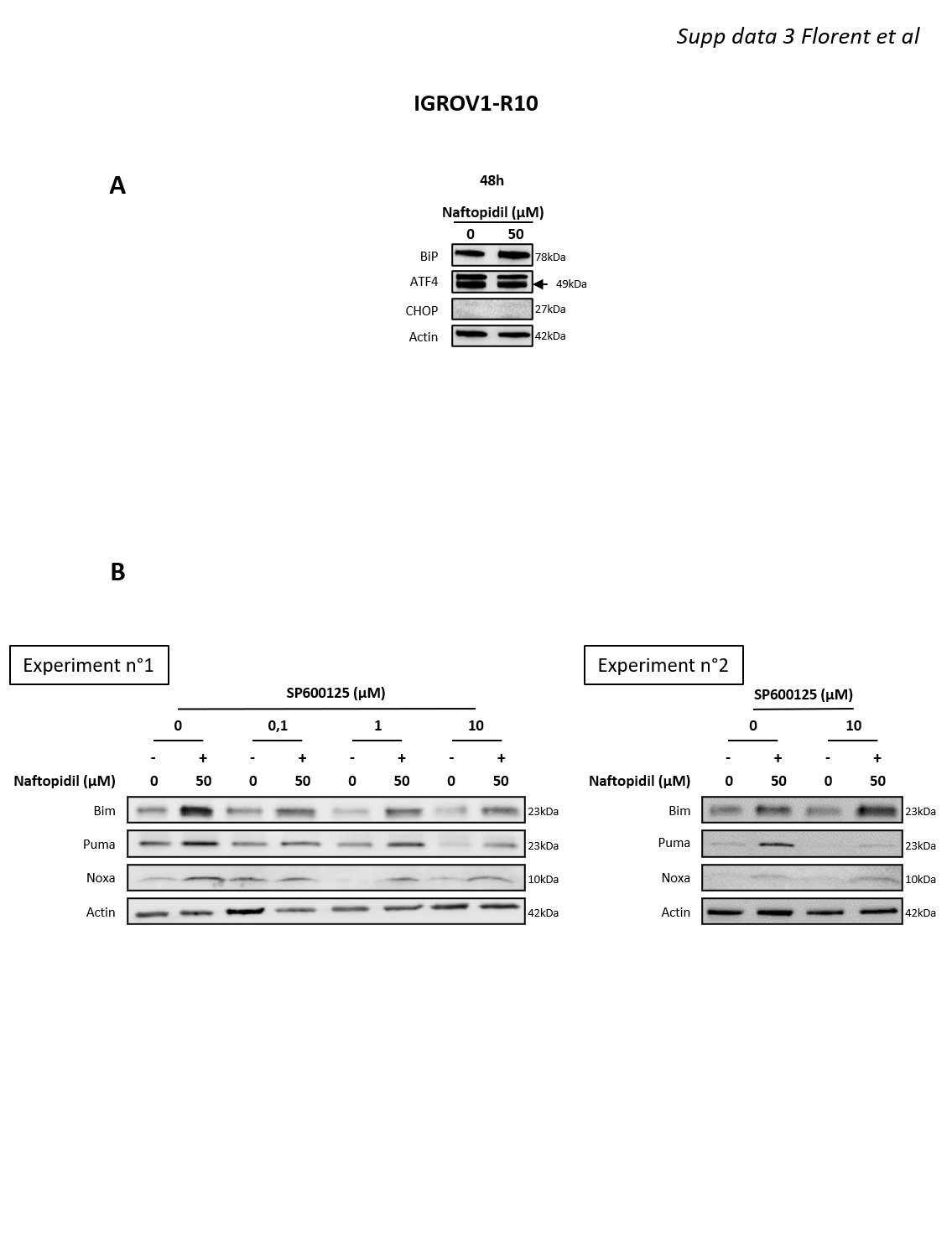

Supplement: Supplementary file 4 — Supplementary data S3 [file 41419_2020_2588_MOESM4_ESM.png]

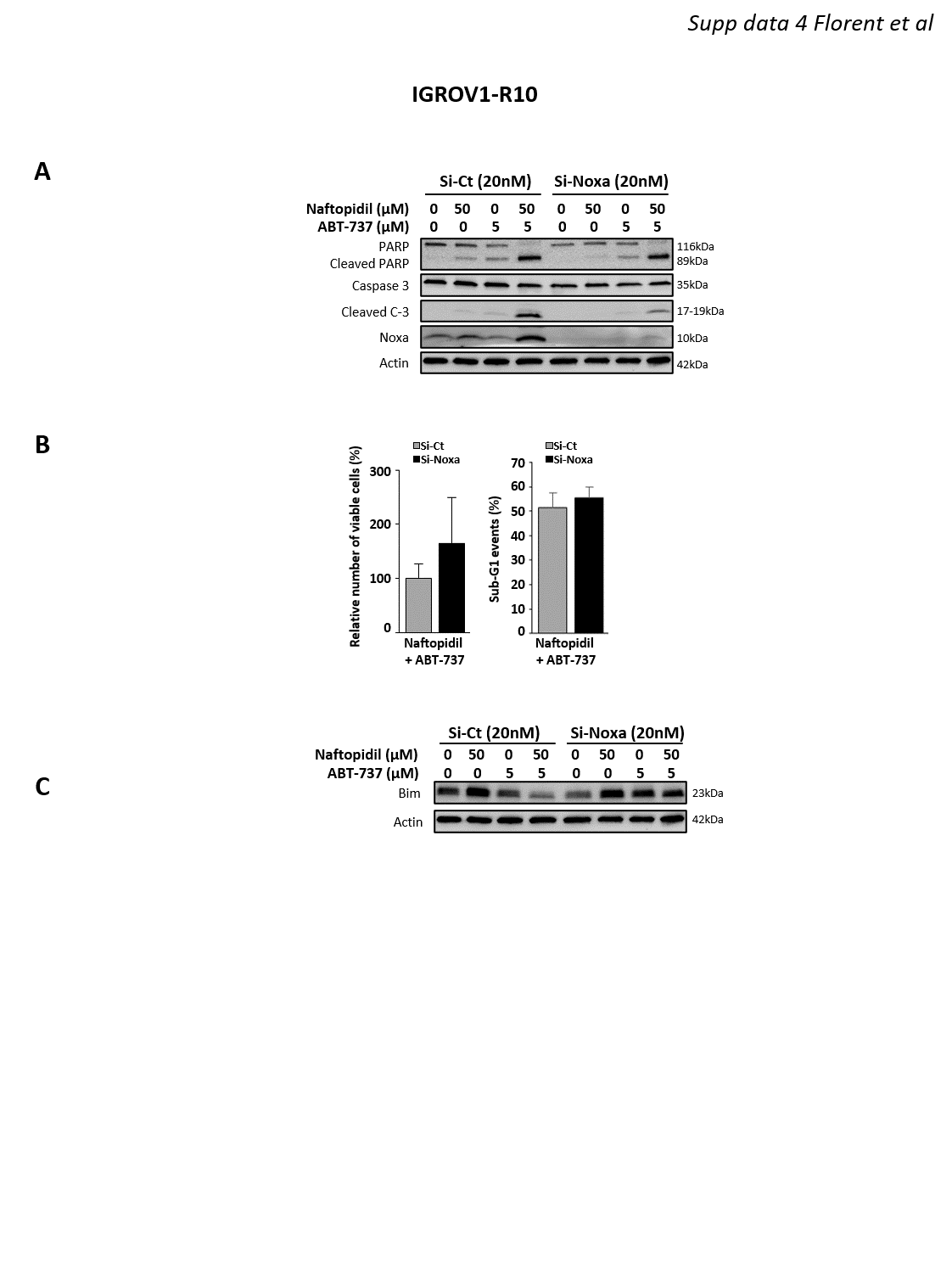

Supplement: Supplementary file 5 — Supplementary data S4 [file 41419_2020_2588_MOESM5_ESM.png]

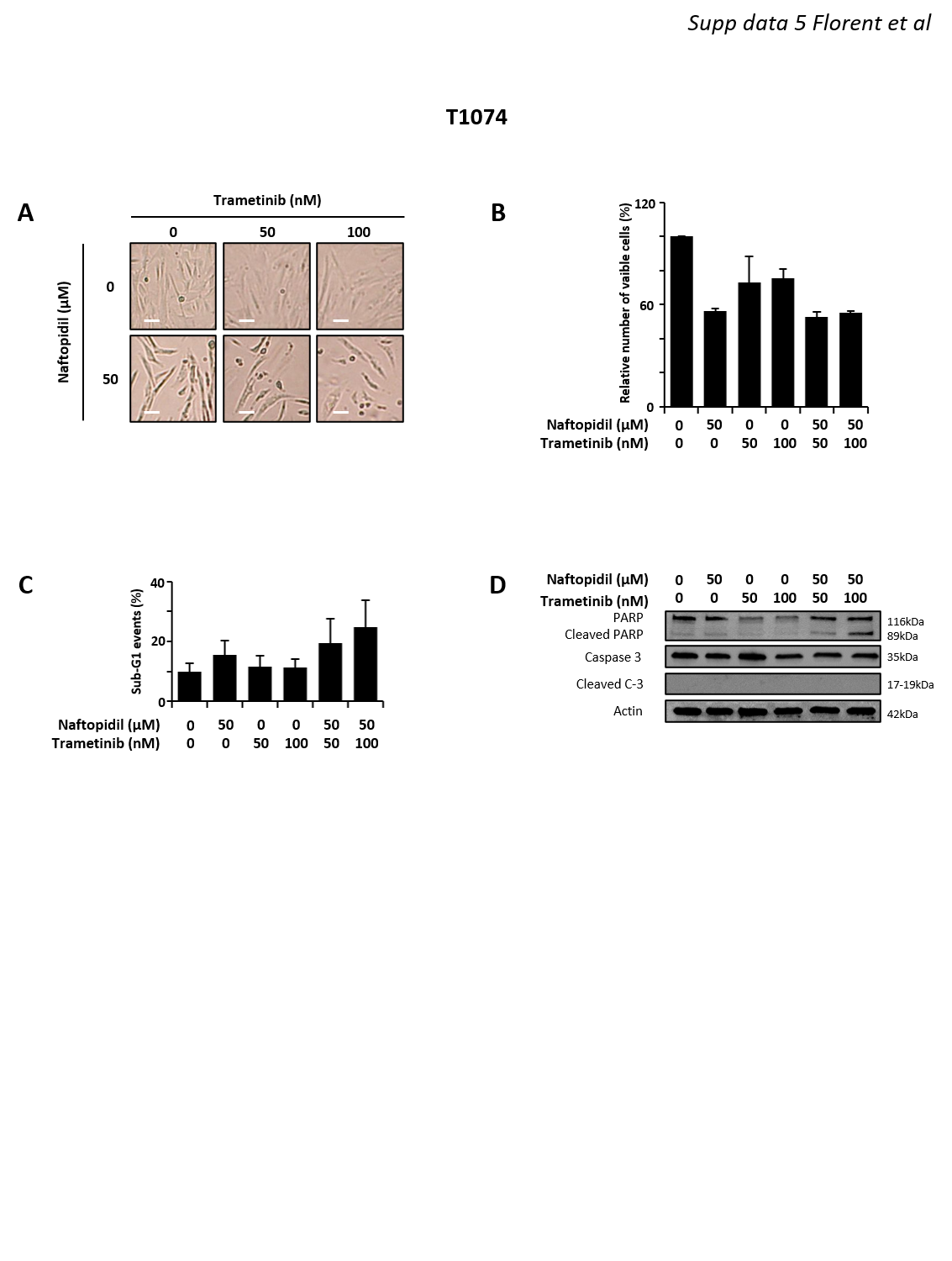

Supplement: Supplementary file 6 — Supplementary data S5 [file 41419_2020_2588_MOESM6_ESM.png]

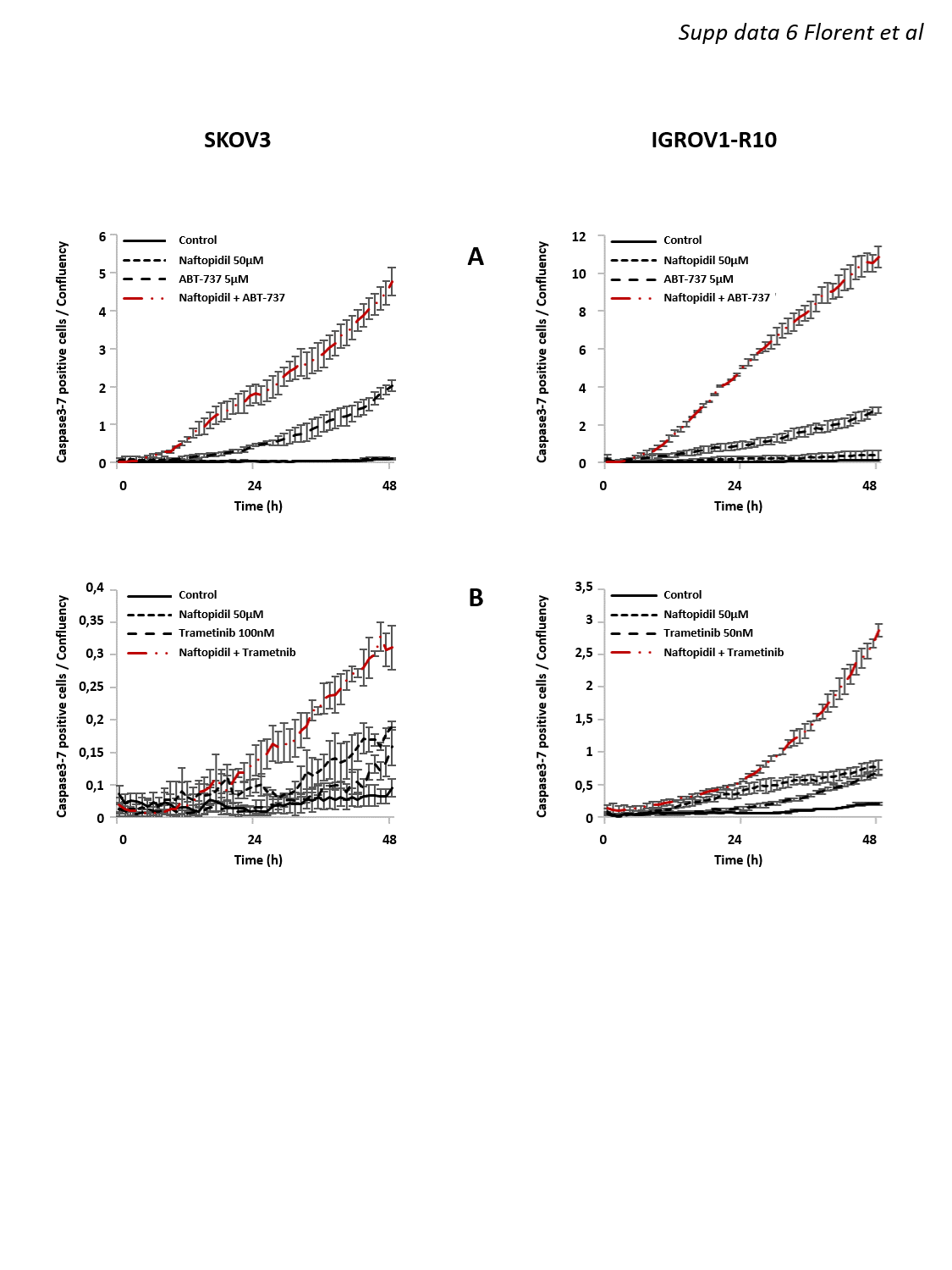

Supplement: Supplementary file 7 — Supplementary data S6 [file 41419_2020_2588_MOESM7_ESM.png]

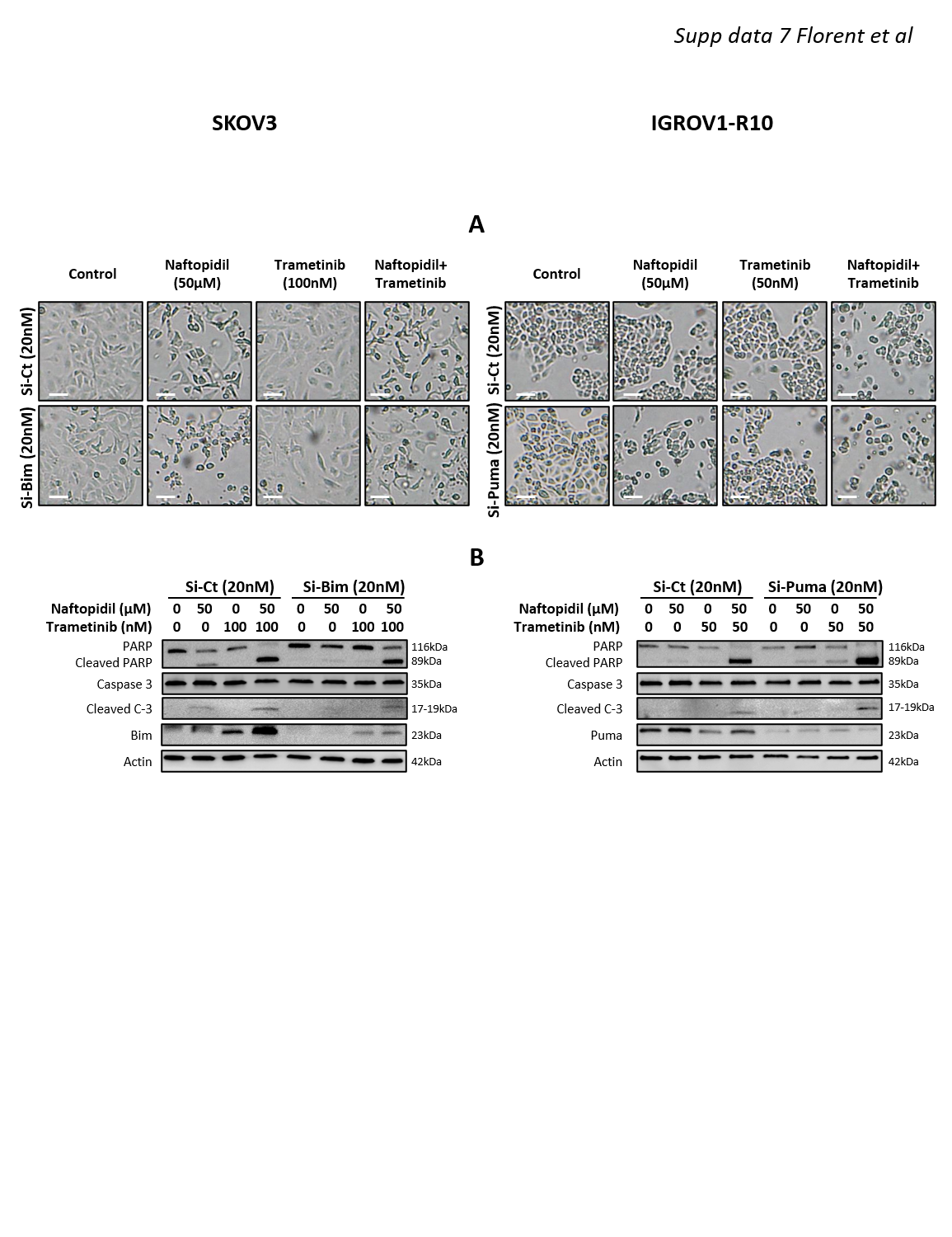

Supplement: Supplementary file 8 — Supplementary data S7 [file 41419_2020_2588_MOESM8_ESM.png]

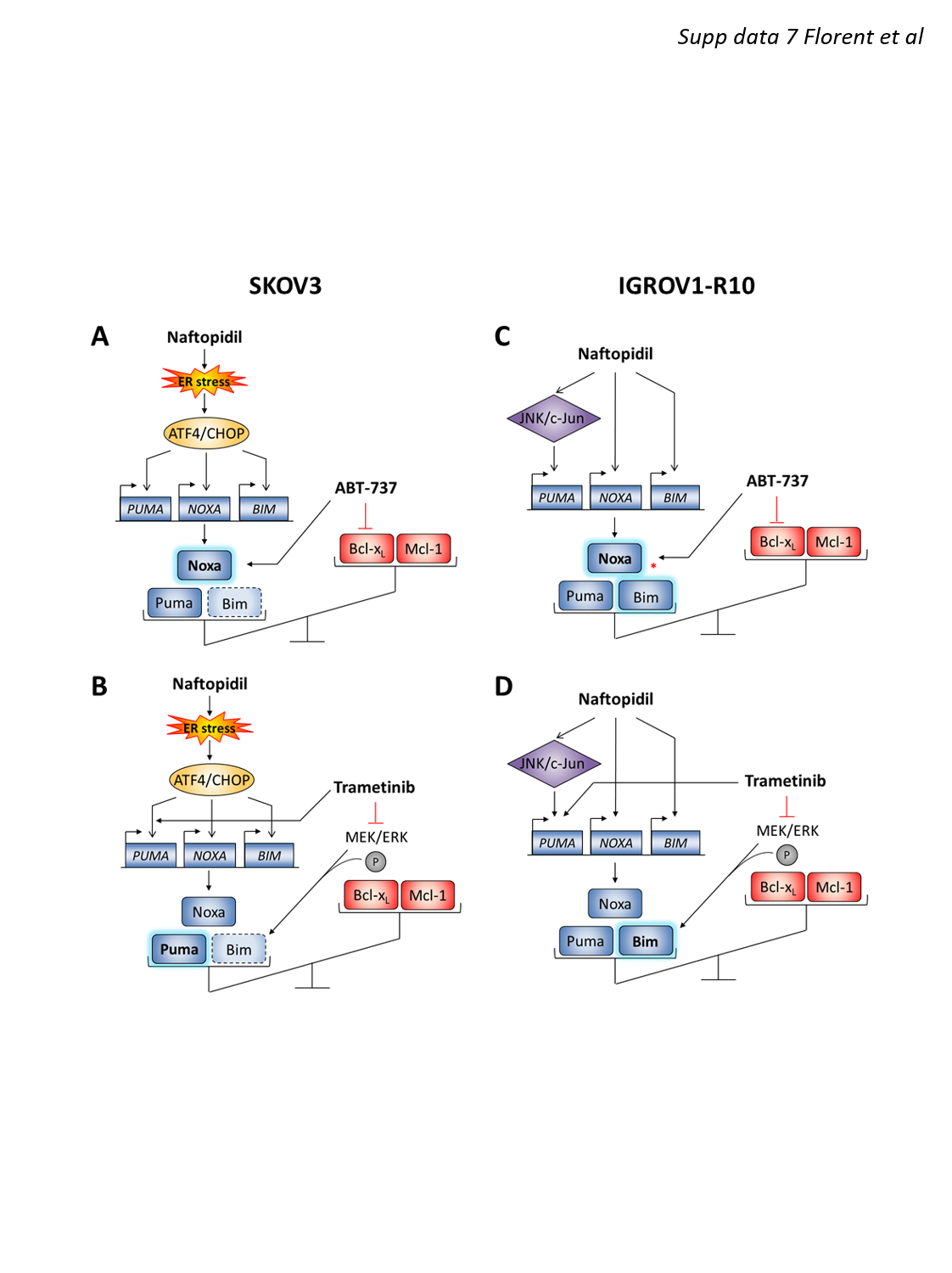

Supplement: Supplementary file 9 — Supplementary data S8 [file 41419_2020_2588_MOESM9_ESM.png]
